# Limitations in the clinical translation of mesenchymal stromal cells: standardisation, heterogeneity and the recipient

**DOI:** 10.3389/fcell.2026.1824475

**Published:** 2026-04-30

**Authors:** Anna Etzenberger, Diana Hernandez

**Affiliations:** 1 Anthony Nolan Research Institute, Immunotherapy, London, United Kingdom; 2 Cancer Institute, University College London, London, United Kingdom

**Keywords:** cell heterogeneity, cell therapy, clinical trials, immunology, Mesenchymal stromal cells

## Abstract

The therapeutic efficacy of Mesenchymal Stromal Cells (MSCs) has been demonstrated in a multitude of pre-clinical studies and clinical trials, yet the translation of MSC therapies is limited by biological factors such as inter- and intra-donor variability in the function, differentiation capacity and proliferation rate of MSCs, as well as extrinsic factors such as the implementation of standardised reporting and characterisation guidelines. The complex relationship between MSC heterogeneity and recipient heterogeneity further contributes to the challenging interpretation of results and complicates the comparison of outcomes between studies. Efforts to enhance the therapeutic efficacy of MSCs have been made, however only a few focus on addressing heterogeneity. Aside from this, a clear understanding of the mechanism of action of MSCs and the development of appropriate functional/potency assays could aid in the standardisation of MSC therapies and provide information for the stratification of recipients. This review discusses the challenges associated with the clinical translation of MSCs and critically evaluates recent advancements in the strategies to enhance the therapeutic efficacy of MSCs.

## 1 Background

Over the last 2 decades, Mesenchymal stromal cells (MSCs) gained intense attention as a novel promising cell therapy due to their unique therapeutic properties for applications in regenerative medicine (Lee et al., 2014; Lim et al., 2021) as well as the treatment of inflammatory diseases (Davies et al., 2025; Etra et al., 2025). MSCs are self-renewing, multi-potent cells with a spindle-like morphology which can be obtained from various types of tissue such as the bone marrow (BM), umbilical cord/Wharton jelly (UC), adipose tissue (AT), placenta/amnion, synovial tissue, peripheral blood, dental follicles, menstrual blood and can be differentiated from iPSCs (Renesme et al., 2025). MSCs are particularly attractive for use in transplant medicine as they are considered immune-privileged due to their low/lack of expression of MHC class I and II molecules as well as co-stimulatory molecules such as B7-1 (CD80), B7-2 (CD86), CD40, and CD40 ligand which play an important role in T-cell activation (Ge et al., 2010; Ge et al., 2009; Zhou et al., 2006). Several pre-clinical studies have been conducted, providing comprehensive information regarding the immunomodulatory efficiency and regenerative properties of MSCs and are extensively reviewed by Spees et al. (2016). Many of these have investigated and demonstrated the vast therapeutic potential and safety of MSCs in the treatment of respiratory diseases (Pang et al., 2021), skeletal pathologies (Lim et al., 2021), inflammatory diseases/immune disorders (Etra et al., 2025), diabetes (Mathur et al., 2023), cardiovascular diseases (Lee et al., 2014) and neurological diseases (Doeppner et al., 2015).

However, although there are 1,850 MSC trials recorded on clinicaltrials.gov (‘Mesenchymal Cells’ 03 February 2026) (ClinicalTrials.gov., 2026), only 11 MSC-based therapies received regulatory approval (Lu and Allickson, 2025). The disproportionate number of clinical studies compared to the number of approved therapies could imply that the therapeutic value of MSCs has been overestimated. Their initial clinical success in GvHD sparked intense interest in the field which meant that MSCs prematurely entered clinical trials for a wide range of applications before their basic biology and mechanism of action were known which has since led to practical discrepancies (Martin et al., 2019). Yet, the vast majority of studies provide supporting evidence for the cells’ therapeutic potential and multiple MSC products have been approved despite the need for a clear description of the MOA of Advanced Therapy Medicinal Products (ATMPs) such as MSCs. This suggests that it is not the therapeutic value of the cells that hinders their clinical translation but rather a lack of standardisation between trials as well as release criteria. Nonetheless, it must be considered that MSCs may not be suitable for all clinical indications which they have been tested for.

Out of the 11 approved MSC therapies, the majority are bone marrow derived (BM-)MSCs, whereas only two commercially available MSC products are adipose derived (Lu and Allickson, 2025). Cartistem (Medipost Co. Ltd., NCT01626677) and Ruibosheng/Amimestrocel (Platinum Life Excellence, NCT07400328) are the only approved umbilical cord derived MSC therapies worldwide (Park et al., 2017). Despite Cartistem’s consistent clinical success in the treatment of repetitive and/or traumatic cartilage degeneration in South Korea since its approval by the MFDS (Ministry of Food and Drug Safety) in January 2012, it is yet to be approved in other countries (Lim et al., 2021). Currently, 9 of the 11 commercially available MSC products are approved in Asia (South Korea MFDS, Japan PMDA, India CDSCO) (Park et al., 2017; Murata et al., 2021; Gupta et al., 2023; Han et al., 2025; Kim et al., 2018; Fernández-Garza et al., 2023; Nam et al., 2022; Wu and Wu, 2024; NMPA, 2025; MFDS, 2017; NC, 2018) while Ryoncil/Remestemcel-L (Kurtzberg et al., 2020; FDA, 2025; Fda and Cber, 2024) is the only FDA (United States) approved MSC product, and MesestroCell (Shahrbaf et al., 2023) is the only commercially available MSC therapy approved in Iran (IR-FDA) (Table 1). Alofisel/Darvadstrocel (Fousekis et al., 2024) received regulatory approval in Europe (2018) and Japan (2021), making it the only approved MSC product in Europe. However, following a randomised, placebo controlled clinical trial in 2023, which revealed that the therapy did not meet the primary clinical endpoint, Alofisel was discontinued (Han et al., 2025; Fernández-Garza et al., 2023).

**TABLE 1:** Approved MSC therapies.

Product	Company	Country	Year	Source	Indication
Temcell HS. Inj	JCR Pharmaceuticals	Japan	2015	BM-MSC	acute graft-versus-host disease (GvHD), Crohn’s disease (Murata et al., 2021)
Stempeucel	Stempeutics Research	India	2016	BM-MSC	Buerger’s disease (thromboangitis obliterans) (Gupta et al., 2023)
Alofisel/Darvadstrocel	TiGenix/Takeda	Europe	2018	AT-MSC	complex perianal fistulas in Crohn’s disease patients (Fousekis et al., 2024)
Stemirac	Nipro Corp	Japan	2018	BM-MSC	spinal cord injury (NC, 2018)
Ryoncil/Remestemcel-L	Mesoblast	Canada/US	2024	BM-MSC	steroid-refractory acute GvHD in children (Fda and Cber, 2024)
Cellgram-AMI	Pharmicell	South Korea	2011	BM-MSC	acute myocardial infarction (Kim et al., 2018)
Cupistem	Anterogen	South Korea	2012	AT-MSC	Crohn’s fistula (Fernández-Garza et al., 2023)
Cartistem	Medipost	South Korea	2012	UCB-MSC	knee cartilage defects (Park et al., 2017)
NeuroNata-R	Corestem	South Korea	2014	BM-MSC	Amyotrophic Lateral Sclerosis (ALS) (Nam et al., 2022)
MesestroCell	Cell Tech Pharmed	Iran IR-FDA	2018	BM-MSC	Approved for various indications (Han et al., 2025; Shahrbaf et al., 2023)
Ruibosheng/Amimestrocel	Platinum Life Excellence	China NMPA	2025	UC-MSC	aGvHD (Wu and Wu, 2024)
Queencell	Anterogen	Korea	2010	heterogeneous mixture	connective tissue disorders (MFDS, 2017)

Both autologous and allogeneic MSC therapies have been approved, highlighting that both can be efficacious and safe. Yet, there are limitations and advantages to both, although allogeneic MSCs are often preferred due to their enhanced function, availability and cost effectiveness (Durand and Zubair, 2022). Particularly since the concerns regarding the immunogenicity of MSCs are minimal, the use of allogeneic MSCs is considered unproblematic. Furthermore, it is well established that MSCs function on a hit-and-run mechanism and therefore do not require persistence in the body in order to execute their therapeutic function. This means that the rejection of MSCs by the host’s immune system in an allogeneic setting is not associated with risks or reduced efficacy. Furthermore, allogeneic MSCs can be available on demand due to large scale manufacturing of off-the-shelf MSC therapies whereas autologous products are manufactured specifically for an individual which can be laborious, cost ineffective and may delay treatment. Therefore, the advantages of using allogeneic MSCs outweigh those of using autologous MSCs (Durand and Zubair, 2022; Wilson et al., 2021).

In Europe, like in the US, MSCs are considered ATMPs and are subject to complex regulations which require compliance with current good manufacturing practice (cGMP) standards. According to the European Medicines Agency (EMA) and Food and Drug Administration (FDA), each part of the manufacturing process including the isolation, expansion and validation of MSCs must adhere to these guidelines and the regulations further extend to the training and qualifications of the scientists handling the products as well as the reagents, controls and facilities used for the production of an MSC product. Additionally, ATMPs must be tested for microbial, endotoxin and *mycoplasma* contamination, viability, clonogenicity, identity and purity as well as potency as these are essential release criteria. However, the lack of standardisation and incoherence in the manufacturing protocols which include, but are not limited to, the culture media composition, potency assessment, MSC source, cell isolation and characterisation between facilities results in heterogeneity between products and ultimately affects clinical outcomes (Sanz-Nogués et al., 2021; Sensebé et al., 2013; Česnik and Švajger, 2024).

**FIGURE 1:**
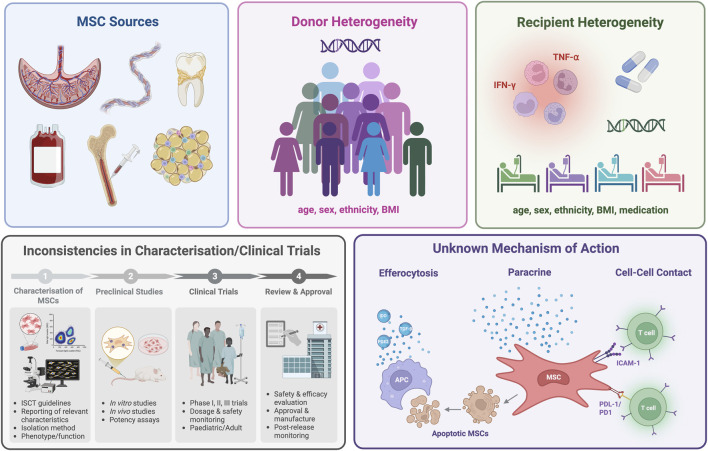
Challenges in the clinical translation of MSCs. Created with BioRender.com.

The limited clinical translation of MSC products despite the enormous clinical potential and research interest highlights the complexity of standardising MSC therapies (Wright et al., 2021). One of the main factors hindering the translation of MSC products is the heterogeneity in the function between MSCs from different sources and donors (Davies et al., 2025; Li et al., 2023) although other factors such as the variability in their proliferative capacity, phenotypic marker expression, differentiation potential, immunosuppressive capacity and secretome can also majorly limit their clinical translation and prevent regulatory approval (Renesme et al., 2025; Wright et al., 2021; Li et al., 2023; Hass et al., 2011; Costa et al., 2021; FDA, 2020). Furthermore, the lack of complete understanding of the mechanism of action (MOA) of the cells complicates the assessment of their potency and prevents the accurate prediction of the therapeutic function of MSCs derived from different donors (Wright et al., 2021; Simon et al., 2024; Patel et al., 2025; Dominici et al., 2006). It is also important to consider the recipient’s immune system when evaluating the function of MSCs as the age and sex of the recipient of the MSC therapy, among other factors, can affect the efficacy of the cells and contribute to the heterogeneity in clinical trial outcomes (Wright et al., 2021; Patel et al., 2025) (Figure 1).

Heterogeneity in clinical trials raises doubt regarding the efficacy of MSC therapies, leading to stagnation of their translation which is exacerbated by the lack of knowledge regarding their mechanism of action, preventing the accurate and reproducible assessment of their potency (Sanz-Nogués et al., 2021; Sensebé et al., 2013; Česnik and Švajger, 2024). Therefore, the standardisation of characterisation and reporting guidelines, manufacturing protocols as well as clinical trial protocols together with high compliance to such guidelines could improve the translatability of MSC therapies. Particularly since the safety and therapeutic efficacy have been tested extensively and are widely accepted, the slow progression from ‘bench-to-bedside’ may not be attributed to the biology/potency of the cells but rather to bureaucratic and standardisation challenges as well as the stratification of patients. Importantly, more extensive research is required to elucidate the MOA of MSCs as it could help scientists and clinicians measure distinct characteristics associated with their MOA and correlate them to clinical outcomes. Therefore, identifying how exactly MSCs achieve their therapeutic function should be prioritised as it could not only improve donor selection and patient stratification but also provide valuable information on how the cells could be manipulated (*via* genetic manipulation or media composition, etc.) to enhance their therapeutic efficacy. This review critically evaluates the factors which hinder the clinical translation of MSCs and discusses recent developments in the approaches to enhance the therapeutic efficacy of MSCs.

## 2 Standardised characterisation methods are required

In order to reduce inconsistencies in the assessment of MSCs between different studies, essential criteria to characterise MSCs were first established in 2006 by Renesme et al. (2022) which resulted in the publication of the minimal criteria for defining multipotent MSCs set by the International Society for Cell and Gene Therapy (ISCT). The initial key criteria included: 1) adherence to plastic, 2) positive marker expression of CD105, CD73 and CD90 in >95% of the MSC population and a lack of expression (<2% positive) of CD45, CD34, CD14 or CD11b, CD79a or CD19 and HLA class II, and 3) tri-lineage differentiation capacity (osteoblasts, adipocytes and chondroblasts) (Renesme et al., 2022). However, due to rapid advances in the field, these criteria have required continuous re-assessment.

The most recent three-round survey using a Delphi method-based approach gathered consensus from 87 subject matter experts from 22 countries on the reporting and characterisation guidelines with the aim to improve transparency and reproducibility for the appropriate assessment of MSC therapies (Renesme et al., 2025). Among the plethora of newly proposed items for the minimal criteria to define MSCs, nine were deemed essential to include. Some of the new criteria emphasise the importance of reporting the positive and negative markers which are used to define the MSCs as well as the % marker expression that is considered the threshold for positive or negative expression by flow cytometry. It has been agreed that there is currently no MSC specific marker for the characterisation of the cells due to their heterogenous nature, however, the recommended positive cell markers for the characterisation of MSCs include CD73^+^, CD90^+^, CD105+ and it was emphasised that a lack of CD45 expression is crucial. Any additional markers used for the characterisation must be reported in order to ensure reproducibility and transparency. Furthermore, cells that meet the minimal criteria should be referred to as “Mesenchymal Stromal Cell” unless stemness can be demonstrated in which case “Mesenchymal Stem Cell” is an appropriate term to report (Davies et al., 2025; Renesme et al., 2025).

However, despite extensive efforts to standardise the characterisation and reporting guidelines for MSC research, many studies fail to report essential information regarding the phenotyping, culture conditions, cell manufacturing process and ‘fitness’ of the administered MSCs which often refers to the cell’s therapeutic efficacy post cryo-preservation (Moll et al., 2014). The use of cryopreserved MSCs is controversial as several pre-clinical studies report impaired immunosuppressive capacity and low responsiveness to stimulation with pro-inflammatory cytokines post-thaw (Marquez-Curtis and Elliott, 2024). Marquez-Curtis and Elliott (2024) report a reduced response rate in patients treated with cryopreserved MSCs compared to patients treated with freshly harvested cells, yet most clinical trials rely on the cryopreservation of the MSC product prior to administration. Since the use of freshly isolated cells is not feasible in the majority of cases, cryopreservation is widely accepted (Chinnadurai et al., 2016), however, it is essential to report the fitness of the cells post cryopreservation and assess changes in their potency post-thaw (Davies et al., 2025; Robb et al., 2024).

Moll et al. (2014) highlight that only 18% of the 1,053 included MSC articles in their study refer to the ISCT guidelines and 80% lacked the description of a functional assay. The lack of adherence to the essential criteria results in inconsistent results across studies, preventing quality assessment and clinical translation (Moll et al., 2014). Failure to report culture conditions (oxygen levels, 2D/3D cultures, licensing), isolation method, MSC viability, MSC dose, functional assays and the tissue source is particularly problematic as these factors can significantly impact the biological function of MSCs which is important to consider when developing cell therapies (Moll et al., 2014; Payne et al., 2025). Until the adherence to ISCT guidelines and transparency regarding the characterisation of MSCs improves significantly, the translation of MSC products will remain challenging as it causes complications in the quality control and comparisons between studies (Moll et al., 2014).

## 3 Mechanism of action remains to be understood

The importance of demonstrating the MSCs’ ‘critical quality attributes (CQAs) (Davies et al., 2025)’ using *in vitro* potency/functional assays was highlighted by Renesme et al. (2025), although it was acknowledged that the exact mechanism of action (MOA) is unknown. The ambiguity regarding the MOA of MSCs further complicates their clinical translation as the demonstration of potency assays is a requirement for FDA approval of cell therapy products (Wright et al., 2021; Simon et al., 2024; Patel et al., 2025; Dominici et al., 2006). Particularly in the context of immune disorders, the assessment of MSC potency is challenging as the inflammatory conditions can affect the function of MSCs (Cheung et al., 2023), resulting in inconsistent clinical responses to the therapy (Galleu et al., 2017; Mesoblast, 2025). It is important to consider that *in vitro* functional assays are not necessarily predictive of the therapeutic efficiency *in vivo* (Patel et al., 2025). Yet, functional *in vitro* assays are important as the design and success of clinical trials strongly depend on the understanding of the MOA, especially since it can vary between different clinical indications (Davies et al., 2025; Martin et al., 2019; Payne et al., 2025).

Following Mesoblast’s biologics license application (BLA) for the approval of RYONCIL (remestemcel-L-rknd), a BM-MSC therapy for the treatment of paediatric steroid refractory acute Graft versus Host disease (SR-aGvHD) in 2020, an additional randomised controlled study to assess its efficiency in adults and/or children as well as a demonstration of the relationship of potency and the biologic activity of the cell therapy was requested by the FDA (2018). The phase III trial included 54 paediatric grade III/IV SR-aGvHD patients and showed that there was a 25.4% (p = 0.0003) increase in the overall response (OR) at day 28 and improved survival rate by day 100 (74% vs*.* 57%) in the treatment group, compared to the matched control group which included individuals who received gold standard therapies such as rituximab. Furthermore, durability of the therapy was measured as part of a 4-year survival study which showed 67% survival at 6 months, 63% at 1 year, 51% at 2 years, and 49% survival by 4 years in patients with an estimated 2-year survival of 25%–38% with the use of gold standard therapies (Weiss and Dahlke, 2019). Due to the compelling clinical results, RYONCIL (Remestemcel-L-rknd) received FDA approval for the treatment of paediatric patients in 2024 after meeting the primary endpoints and safety requirements (Weiss and Dahlke, 2019). However, despite its clinical success, the mechanism of action of RYONCIL remains unclear. *In vitro* studies show that MSCs have immunomodulatory functions, measured by their ability to suppress T-cell activation/proliferation, polarise monocytes/macrophages toward an anti-inflammatory phenotype, and secrete immunomodulatory factors. This data is consistent with clinical data which shows a 64% reduction in activated T-cells (CD3^+^CD4^+^CD25+HLA-DR+) post treatment. The *in vivo* efficacy was assessed using blood samples of paediatric patients with SR-aGvHD (n = 40; age range 0.6–17 years) were analysed which showed a 79% reduction in tumour necrosis factor receptor type I (TNFR1) and 75% reduction in suppressor of tumorigenicity 2 (ST2) after treatment with RYONCIL compared to the baseline levels at day 180 which are similar to the levels observed in GvHD.

Multiple potential MOAs have been described in the context of various target diseases and MSC sources. The proposed mechanisms of action of MSCs have been described in detail elsewhere (Patel et al., 2025; Ren et al., 2010) however, briefly, commonly suggested MOAs include the secretion of paracrine factors such as indoleamine 2,3-dioxygenase (IDO), prostaglandin E2 (PGE2), transforming growth factor beta (TGF-β), interleukin-6 (IL-6), HLA-G, and the direct cell-to-cell contact dependent mechanism via various targets such as T-cell chemokine ligand (CXCL), intercellular adhesion molecule 1 (ICAM-1), vascular cell adhesion protein 1 (VCAM1) and inhibitory molecule programmed death 1 (PD-1) (Hodgson-Garms et al., 2025; Yen et al., 2020; Davies et al., 2017; Maged et al., 2024). However, an alternative MOA has been proposed whereby apoptotic MSCs are efferocytosed by recipient monocytes/macrophages and dendritic cells (Cheung et al., 2023), and subsequently secrete anti-inflammatory factors including IDO, PGE2, IL-6 and programmed death-ligand 1 (PDL1) to exhibit their immunomodulatory effects (Galleu et al., 2017).

Mesoblast (2025) and Galleu et al. (2017) demonstrate that MSCs undergo apoptosis upon contact with PBMCs from recipients who have an inflammatory immune disorder (GvHD/Crohn’s), but not healthy donor PBMCs, suggesting that the activation of immune cells, as is the case in aGvHD, is essential for the ‘efferocytosis’ MOA. Furthermore, Pang et al. (2021) state that the condition of MSCs at the time of administration appears to have a significant impact on their therapeutic value as neither genetically modified MSCs which were unable to die by apoptosis (BAK/BAX KO) nor heat-inactivated/fixed MSCs were able to achieve comparable anti-inflammatory effects to apoptotic MSCs in their mouse model, likely due to the changes in the MSCs’ metabolic function, secretome and proliferative capacity (Pang et al., 2021; Ren et al., 2010). It was also highlighted in the study that the majority of MSCs injected into BALB/c, NSG or BRGS mice underwent apoptosis and became undetectable within 3 days, yet their therapeutic function did not diminish upon their disappearance (Pang et al., 2021). Interestingly, the authors propose that the therapeutic action of MSCs is dependent on the phagocytic clearance of the apoptotic MSCs, as opposed to cell-intrinsic immunosuppressive properties of viable MSCs. Furthermore, apoptotic MSCs did not directly inhibit T cell proliferation in their *in vitro* assays which further emphasises the importance of phagocytes in this context (Pang et al., 2021). The lack of standardised potency assays that reflect the *in vivo* fate of MSCs prevents the accurate prediction of the therapeutic function of MSCs from different donors. A complete understanding of the MSCs’ MOA would support the development of potency assays as they could be tailored to measure distinct characteristics of the MOA allowing for the reliable assessment of the functional properties of each MSC batch. It is also possible that there are multiple mechanisms associated with the potency of MSCs and their relevance may depend on the application (i.e., regenerative medicine or immune disorders, etc.) in which case several potency assays could be accepted by regulatory authorities (Wright et al., 2021; Patel et al., 2025). Nevertheless, efforts to reduce donor heterogeneity have been made in order to achieve more reliable and consistent therapeutic outcomes, however, more research is required to develop appropriate solutions (Wright et al., 2021; Patel et al., 2025).

## 4 MSC heterogeneity is a limitation for their clinical translation

Significant variability is not only observed in the study design and reporting of important characteristics but there is also biological heterogeneity between MSCs from different donors and tissue sources which complicates the development of MSC therapies (Phinney, 2012). These differences manifest in the function, secretome and differentiation capacity of the cells (Phinney, 2012; Pires et al., 2016; Zhang C. et al., 2021). The heterogeneity not only applies to MSCs from different donors (inter-donor heterogeneity) but also MSCs from one individual (intra-donor heterogeneity) (Phinney, 2012; Kolliopoulos et al., 2023). Therefore, it is recommended to disclose relevant donor characteristics (*i.e.,* age, sex, ethnicity) as well as the tissue source (bone marrow, umbilical cord tissue/blood, adipose tissue, *etc.*) from which the MSCs are obtained, as these factors can influence the phenotype and function of the cells (Renesme et al., 2025; FDA, 2020). There are no limitations on the type of tissue that can be used to obtain the cells as long as they are well characterised (Renesme et al., 2025; Phinney, 2012; Jin et al., 2013).

MSC were first isolated from bone marrow which has since been the most commonly used source of MSCs (Dominici et al., 2006), however current research is becoming increasingly invested in MSCs from adipose tissue and umbilical cord material (Wright et al., 2021). In the clinical setting, the majority of the approved MSC therapies are adult tissue derived (Mello et al., 2024), possibly due to their application in regenerative medicine since AT-MSCs and BM-MSCs seem to have an improved osteogenic differentiation capacity compared to UC-MSCs (FDA, 2020). Many research groups have attempted to determine specific tissue dependent biological differences, however, the results can be contradictory and incomparable as extensively reviewed by Li et al. (2023) and Nemeth (2014). The advantage of UC-MSCs is that they can be obtained via non-invasive methods, posing minimal risk to the donor whereas MSC extractions from bone marrow and adipose tissue can be associated with complications (Costa et al., 2021; Ren et al., 2010).

Kolliopoulos et al. (2023) demonstrated that the transcriptome between MSCs from different tissue sources varies significantly, particularly affecting genes associated with cell adhesion, cell proliferation, cytokine signalling, wound healing, and organ development. The authors therefore suggest that MSCs from different sources could have specific benefits for particular applications (Kolliopoulos et al., 2023). Furthermore, the study highlights that differences in the gene expression are also observed in MSCs isolated form one tissue source (referred to as ‘intra-tissue MSC subpopulations’), leading to heterogeneity in the immunomodulatory capacity of MSCs isolated from the same tissue source, measured by their ability to suppress BV2-cells in a neuro-inflammation model (Kolliopoulos et al., 2023). Interestingly, NF*κ*B and interferon response transcription factor binding sites were positively correlated with immune suppression in their model, indicating that these pathways could play a major role in the function of MSCs in the context of immune modulation (Kolliopoulos et al., 2023). Additionally, MSC stimulation with pro-inflammatory cytokines (IFN-γ and TNF-⍺) changed the cells’ transcriptome but did not eradicate differences in the transcriptome between donors as the authors initially hypothesised (Kolliopoulos et al., 2023).

Multiple factors contribute to the vast heterogeneity that is observed between donors (Francis et al., 2010). Age, sex, genetic factors, BMI, chronic disease and potentially unknown factors affect the proliferative capacity, function and potency of MSCs (FDA, 2020). Although the effects of obesity on the therapeutic quality of MSCs has not been studied in detail, it is an essential factor to consider as many adipose tissue derived MSCs stem from liposuctions performed on obese or overweight donors. Since obesity is associated with inflammation and genetic differences compared to non-obese donors, it is recommended to conduct potency tests for MSCs from each donor to confirm their proper functionality prior to their assessment in clinical trials. Alternatively, AT-MSCs may be obtained from healthy non-obese donors which may be associated with (avoidable) complications although the risks are considered relatively low compared to those associated with bone marrow aspirates (FDA, 2020; Selle et al., 2022). Essential qualities of MSCs also change with increasing age of the donor (FDA, 2020; Wu et al., 2014). Many groups report heterogeneity in the differentiation capacity and potency of MSCs from male and female donors, however these trends are not consistent and vary significantly between each model and tissue type (Phinney, 2012). Yet, it may be important to consider the donor’s sex in clinical trials as differential outcomes may be explained by the donor’s sex among other factors (Phinney, 2012).

Wu et al. (2014) report that there was a significant increase in the doubling time of human BM-MSCs with increasing age in female donors in their study. Interestingly, the authors did not observe this correlation in male donors (Wu et al., 2014). Similarly, the differentiation capacity of aging female donors decreased, whereas in male donors, the MSCs’ ability to differentiate was not affected by age (Wu et al., 2014). This study suggests that aging has a more significant effect on MSCs in female donors compared to male donors which is an important consideration for clinical trials (Wu et al., 2014). Other studies also show that MSCs from older donors have an impaired immunosuppressive capacity compared to younger donors, likely driven by changes in the gene expression and secretome in elderly donors (Mancini et al., 2017; Zhang Y. et al., 2021; Huang et al., 2019). Cheung et al. (2019) demonstrate that MSC-derived extracellular vesicles (MSC-EVs) from a young hAT-MSC donor (age 25 years) induced an anti-inflammatory phenotype in lipopolysaccharide (LPS) stimulated macrophages whereas MSC-EVs from the older AT-MSC donor (age 72) did not (Cheung et al., 2019). Although the data regarding age related variability in the immunosuppressive capacity of MSCs is compelling, further research is required to understand the exact impact and the associated mechanisms that drive the changes which occur in aging donors. Perinatal tissue derived MSCs such as UC-MSCs are not subject to age-dependent variability, making them ideal for the use as a cell therapy although they are not exempt from variation due to other factors (Phinney, 2012).

## 5 MSC interactions with the recipient’s immune system

In addition to the donor related differences, it is also important to consider the MSCs’ interactions with the recipient’s immune system. The varying immunocompetence of the recipient is particularly accentuated in the context of GvHD (Pang et al., 2021; Mesoblast, 2025; Francis et al., 2010; Gong et al., 2025). Gong et al. (2025) emphasise that the recipient’s immune system plays an important role in the activation of MSCs and their subsequent immunomodulatory efficiency which could explain the variability in response rates to MSC treatments in clinical trials as GvHD patients whose PBMCs were more cytotoxic against MSCs had improved response rates to MSC treatment than those with less cytotoxic efficiency. The secretion of pro-inflammatory cytokines, specifically TNF-⍺ and interferon-γ (IFN-γ) by recipient immune cells, appears to be the main driver of the cytotoxicity against MSCs and therefore plays an important role in their activation, similar to the licensing of MSCs (Pang et al., 2021; Mesoblast, 2025; Gong et al., 2025).

Commonly used immunosuppressive drugs such as infliximab and adalimumab, which act by neutralising TNF-⍺, prevent the recipient mediated activation of MSCs, decreasing their immunosuppressive efficiency which ultimately leads to inconsistent results in clinical trials (Pang et al., 2021; Cheung et al., 2023; Mesoblast, 2025; Francis et al., 2010). Other drugs including Glucocorticoids as well as COX inhibitors have been reported to decrease the immunosuppressive capacity of MSCs, adding more obstacles to the clinical translation of MSCs (Francis et al., 2010). *In vitro* studies or animal models can rarely appropriately depict the complex inflammatory milieu of each individual MSC recipient in the context of immune disorders as the recipient-recipient differences can be significant and unpredictable (Francis et al., 2010). As previously mentioned, the phagocytosis of apoptotic MSCs is one of the likely mechanisms by which the cells achieve their therapeutic effects. Therefore, recipients with a suppressed immune system and impaired phagocytes may have an inadequate response to MSC therapy (Gong et al., 2025). Patient stratification may be aided by measuring the immunosuppressive activity of a MSC donor against immune cells of a particular patient/recipient *in vitro* prior to administration in order to assess whether the MSC donor is an appropriate ‘match’ for the recipient (Galleu et al., 2017; Francis et al., 2010). HLA-unrelated differences which may cause incompatibility can be determined so that the most appropriate donor can be chosen, as the prediction of the therapeutic potency of a MSC donor in different recipients is challenging due to the lack of established and reliable biomarkers (Mesoblast, 2025; Gong et al., 2025).

Furthermore, heterogeneous clinical trial outcomes may also be caused by the recipient’s age which is not an extensively studied factor in the context of MSC therapy. This could explain why certain MSC therapies such as Remestemcel-L (Ryoncil) have been more successful in paediatric patients than adults. It is widely accepted that the immune system significantly changes with age and is often associated with increased inflammation, marked by high levels of C Reactive protein (CRP), IL-6, TNF and IL-8, susceptibility to infections and a diminished immune response to vaccines and is therefore frequently referred to as ‘inflammageing’ (De Maeyer et al., 2025; Kuka et al., 2025). Since monocytes are an essential part of the efferocytosis MOA of MSCs, it is important to understand how inflammageing affects this immune component. Kuka et al. (2025) studied the proteome of monocytes in young (<40 years) vs*.* old (≧65 years) females and discovered that most of the studied proteins were downregulated in older females, including proteins involved in the complement cascade such as C3, C4 and C1QBP) as well as GAPDH which plays a role in cell metabolism. Proteins important for migration (CD44, TUBB4B, TUBB and ITGAL) and scavenger receptors (CD68 and CD48) were also downregulated in older females. Interestingly, proteins associated with endocytosis (HGS and EEA1), intracellular signalling (MAP2K4, PPIP5K2), ubiquitination (WDR48, CUL4A), chromatin remodelling (SMARCB1) and intracellular organelle motility (KTN1) were significantly upregulated in monocytes from older females. The authors also report a significant increase in the proportion of intermediate and non-classical monocytes in older adults compared to younger adults. Most importantly, however, phagocytosis-associated pathways which include phagocytosis, and the complement cascade were found to be downregulated in older females which can have a notable impact on the therapeutic function of MSCs since the phagocytic activity of monocytes is essential in order for MSCs to execute their function (Pang et al., 2021; Mesoblast, 2025; Kuka et al., 2025).

Since inflammation plays an important role in the activation and *in vivo* fate of MSCs, the patient’s inflammatory state should be assessed prior to the administration of the therapy as it may provide valuable information regarding the patient’s response to the treatment. Studies assessing the effect of the recipient’s age on the function of MSCs often do not include a wide range of ages which complicates the interpretation of the results. Additionally, the chronological age of an individual may not represent their biological age which could prevent the appropriate stratification of recipients (De Maeyer et al., 2025). In a study, dissecting the immune system of 300 adults (age 25–90 years) over time using proteomics, flow cytometry and single cell RNA sequencing, De Maeyer et al. (2025) found an abundance of transcriptional age-related changes in T cells, and only a few in non-T cells. The majority of the differentially expressed genes were found in naïve T cells, central memory T (T_CM_) cells and effector memory T (T_EM_) and were specifically age related and distinct from the sex related changed that were detected (De Maeyer et al., 2025). This observation could have important implications in the functional assessment of MSCs as their ability to suppress T cells is the gold standard potency assay (Patel et al., 2025). Therefore, the recipient’s age should be taken into account when interpreting the potency of MSCs.

## 6 Strategies to reduce MSC heterogeneity

**FIGURE 2:**
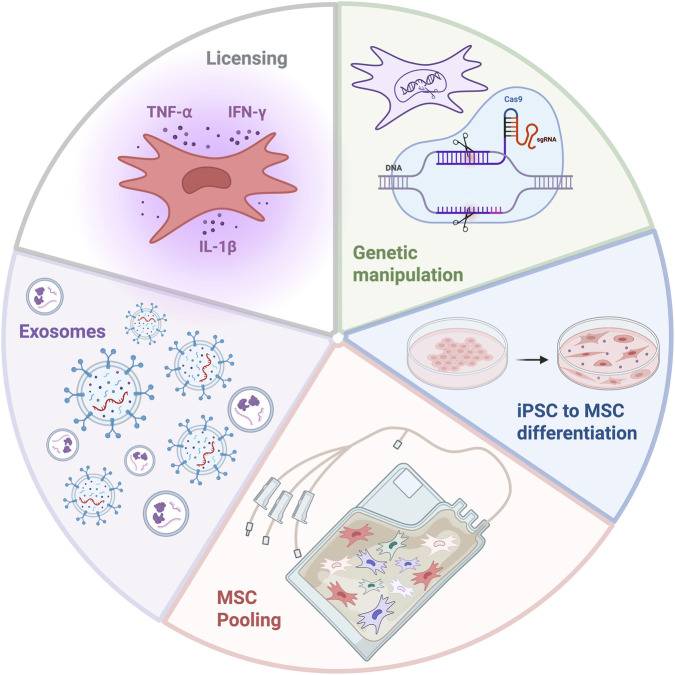
Strategies for the reduction of MSC donor functional heterogeneity and the enhancement of their therapeutic efficiency. Created with BioRender.com

Current attempts to reduce the functional variability between MSC donors and enhance their therapeutic capacity include pooling cells from multiple MSC donors in a single product, priming/activating MSCs using pro-inflammatory cytokines which is also referred to as MSC licensing (activation with pro-inflammatory cytokines), differentiating them from induced pluripotent stem cell (iPSC) synergising MSCs with drugs or other cells and using non cellular MSC derived particles such as extracellular vesicles and proteins, yet none have successfully eliminated functional donor heterogeneity (Dominici et al., 2006; Cheung et al., 2023; Nemeth, 2014) (Figure 2).

Pooling MSCs from different donors of varying fitness has been one of the attempts to overcome functional heterogeneity (Kannan et al., 2024; Hejretová et al., 2020). However, there are challenges in the execution, as the proportion of the pooled cells from different MSC donors can change significantly in each ‘pool’ between passages which results in an inconsistent product and unpredictable efficacy (Hejretová et al., 2020). Although several groups have reported a reduction in the functional variability in pooled samples compared to individual donors, the differences are often not significant or maintained across passages (Kannan et al., 2024; Hejretová et al., 2020; Hansen et al., 2022; Valencia et al., 2025). Indeed, Stempeucel is an approved MSC product which consists of pooled BM-MSCs. However, it has been approved for the treatment of regenerative disorders which requires different properties to the treatment of immunomodulatory disorders. Therefore, further optimisation is required in order to achieve a reduction in the heterogeneity of MSCs through pooling in different disease contexts.

Another common strategy used in the attempt to reduce the heterogeneity in the function of MSCs from different donors is the pre-treatment of the cells with pro-inflammatory cytokines such as TNF-⍺, IFN-γ and/or IL-1β prior to the functional assay or administration (Cheung et al., 2023). This activation method is referred to as ‘licensing’ which sensitises MSCs to apoptosis, enhances their clearance *in vivo*, and causes changes in their secretome which has ultimately been shown to improve their therapeutic function (Pang et al., 2021; Cheung et al., 2023; Galleu et al., 2017; Mesoblast, 2025; Yen et al., 2020; Gong et al., 2025). Although the licensing of MSCs has shown an improved therapeutic efficacy in multiple compelling studies, some groups have demonstrated that the inflammatory milieu present in GvHD, asthma or diabetes, may already provide the appropriate environment to achieve their therapeutic function (Shi et al., 2025). Therefore, it is possible that the activation of MSCs by pro-inflammatory cytokines occurs *in vivo* without the need to prime the cells prior to the administration (Shi et al., 2025).

The genetic manipulation of MSCs is the most novel approach for the reduction of MSC donor functional heterogeneity and the enhancement of their therapeutic efficiency (Nemeth, 2014). While MSCs have previously been genetically modified for a variety of applications including cancer treatment (Xu et al., 2020) by expressing tumour necrosis factor (TNF)-related apoptosis-inducing ligand (TRAIL), IL-10, HGF, FOXP3, and VEGF, to our knowledge, there has been no success in reducing their functional heterogeneity *via* genetic manipulation to date. However, rather than reducing heterogeneity, efforts have been made to enhance the therapeutic effect of MSCs. Dashti et al. (2025) overexpressed the immunosuppressive cytokine IL-37 though lentiviral transduction with the aim to enhance the therapeutic activity of MSCs and found that IL-37-MSCs had improved anti-inflammatory functions and reduced autoantibody production in a systemic lupus erythematosus mouse model compared to treatment with IL-37 or MSCs alone, suggesting that there is an additive effect once the two therapies are combined (Dashti et al., 2025). With the increase in the use of the CRISPR/Cas9 technology, the genetic manipulation of MSCs has become more attractive as it allows for the precise editing of a gene of interest with high efficiency and low cytotoxicity in MSCs (Han et al., 2024; Meng et al., 2019). The current focus of the genetic manipulation of MSCs lies in the reduction of immunogenicity by knocking out the 2-microglobulin (B2M) gene, resulting in the loss of HLA class I expression which is a potential concern in activated MSCs. Meng et al. (2019) show that the B2M KO MSCs have an improved immunosuppressive capacity and survival compared to unedited MSCs. Bloor et al. (2020) used CRISPR/Cas9 to overexpress IL-10 which subsequently prevented inflammatory cell infiltration and the secretion of pro-inflammatory cytokines and improved cardiac functional recovery. Therefore, the genetic manipulation of MSCs can help make significant advancements in the standardisation and therapeutic efficacy of MSC therapies.

Alternative proposed methods to overcome the challenges associated with the standardisation of MSCs include differentiation from induced pluripotent stem cell lines (iPSC) (iMSCs) as iMSCs have unlimited proliferation capacity without the loss of the therapeutic function of tissue-isolated MSCs. The consistency, scalability and lack of batch-to-batch variation are significant advantages of this therapy however, their tumorigenicity and genetic instability remain a major concern of iPSCs and therefore further robust clinical data is required before iPSCs can replace tissue-derived MSCs (Olmedo-Moreno et al., 2022; Kou et al., 2022). MSC-derived extracellular vesicles (EVs) including exosomes and microvesicles are a unique ‘cell-free’ therapy as they are considered an extension of MSCs with almost identical therapeutic functions but without the immunogenicity. Unlike, iPSC derived MSCs, EVs are not associated with tumorigenicity and are considered a safe therapy^91^. Furthermore, they are not subject to genetic instabilities or loss of function due to cryopreservation which may be important for certain applications^91^. Due to their unique ability to cross the blood-brain barrier, EVs have a particular advantage in the treatment of neurological disorders. The low cost of EV production and unproblematic characterisation are additional benefits although EVs have been reported to lack specificity which can be problematic due to off-target effects(Dominici et al., 2006)^,91^. Efforts to enhance their precision and clinical efficacy have been made *via* the overexpression of ligands and addition of therapeutic cargo through electroporation with varying success (Dominici et al., 2006). Unfortunately, despite the advantages of MSC-EVs, functional heterogeneity has also been observed in EVs which does not resolve the problems associated with the standardisation of MSC products(Dominici et al., 2006; Kou et al., 2022)^91^.

## 7 Conclusion

MSCs have vast therapeutic potential across a plethora of diseases, particularly in regenerative medicine and transplant medicine. However, due to inconsistencies likely caused by donor heterogeneity, the uncertainty regarding the mechanism of action, recipient associated variability, and the lack of standardisation of characterisation methods and reporting strategies across pre-clinical studies and clinical trials, the translation rate into clinical products is poor. Despite the efforts made to ameliorate functional donor heterogeneity, a solution has yet to be developed, partially due to the lack of appropriate *in vitro* potency assays which take into consideration the recipient’s immune system. Therefore, innovative approaches to dissect the interactions between the recipient’s immune system and MSCs are required, while accounting for the heterogeneity between MSC donors as well as the recipients. The development of *in vitro* assays which appropriately depict the environment of the recipient is therefore essential as confounding factors which impact the MSC-immune cell interactions are often not considered in current assays. Reducing functional heterogeneity MSCs may result in more consistent clinical outcomes rather than improved therapeutic efficacy of the cells *per se*. A more consistent product would also allow for more consistent and predictable therapeutic results and less variation in clinical trials which may contribute to the translation of MSC therapies as it is important that a therapy has equal efficacy in a wider patient cohort, providing patients are stratified appropriately. If MSCs are manipulated to have enhanced therapeutic properties, it may be possible to improve the potency of less potent donors resulting in less variation and improved clinical outcomes. Most importantly, the adherence to ISCT guidelines is essential as poor implementation leads to inconsistencies between trials and can lead to the misinterpretation of results which ultimately prevents the approval of novel MSC therapies.
